# Cryptococcosis in patients with hematological diseases: a 14-year retrospective clinical analysis in a Chinese tertiary hospital

**DOI:** 10.1186/s12879-017-2561-z

**Published:** 2017-07-03

**Authors:** Rui-ying Wang, Yan-qiong Chen, Ji-qin Wu, Xuan Wang, Ya-hui Cao, Hua-zhen Zhao, Li-ping Zhu

**Affiliations:** Department of Infectious Diseases, Huashan Hospital, Fudan University, 12 Central Urumqi Road, Shanghai, China

**Keywords:** Cryptococcosis, Hematological diseases, Cryptococcal meningitis, Pulmonary cryptococcosis, HIV-uninfected

## Abstract

**Background:**

Cryptococcal infection has become a public health challenge globally. However, information about cryptococcal infection in patients with hematological diseases remains relatively rare.

**Methods:**

HIV-uninfected cryptococcosis cases with hematological diseases admitted to Huashan Hospital from January 2001 to December 2014 were reviewed.

**Results:**

In total, 33 cryptococcosis patients were enrolled, including 12 malignant and 21 non-malignant hematological cases. Twenty-six patients had central nervous system (CNS) involvement, which was observed more often in patients with non-malignancies than with malignancies (20/21 vs. 6/12, *P* = 0.001) Most patients (25/26) with CNS infection were confirmed by cerebrospinal fluid (CSF) culture or smear, and 100% (20/20) of them tested positive for the CSF cryptococcal antigen test. Eighteen out of 26 cryptococcal meningitis patients were treated with amphotericin B (AmB)-based therapy, 16 of them with AmB deoxycholate (d-AmB) and 2 patients with liposomal AmB. The clinical success rate was 55.6%. D-AmB was well-tolerated at 0.35–0.59 mg/kg/d (median 0.43 mg/kg/d) and only 12 patients had mild adverse events.

**Conclusions:**

CNS cryptococcal infection was more frequent in patients with hematological non-malignancies, and cryptococcal antigen test as well as the CSF fungal culture or smear are suggested for early diagnosis. D-AmB could be used as an alternative therapy for CNS-infected patients with hematological diseases.

**Electronic supplementary material:**

The online version of this article (doi:10.1186/s12879-017-2561-z) contains supplementary material, which is available to authorized users.

## Background

Cryptococcosis is a life threatening disease in patients with immunocompromised conditions, such as human immunodeficiency virus (HIV) infection, solid organ transplants (SOT), autoimmune diseases, administration of corticosteroids and other immunosuppressants [1, 2]. Globally, about 957,900 cryptococcal meningitis cases occur each year, resulting in 624,700 deaths by 3 months after infection among HIV-infected patients [3]. Moreover, cryptococcal infection could also occur in 2.8–8.0% of SOT recipients, and is the third most common invasive fungal infection in these patients, after *Candida* and *Aspergillus* infections [4, 5]. However, studies on cryptococcal infections under hematological conditions are rare [6–8]. The SEIFEM-2004 study showed that only 8 cryptococcosis cases were documented among 11,802 patients with hematological malignancies, corresponding to an extremely low incidence of 0.07% [9]. According to a 30-year autopsy study, 340 cases had invasive mycoses among 1591 autopsied patients with hematological neoplasias [10]. Among them, *Aspergillus* and *Candida* accounted for 52.6% and 31.4% respectively, while only 2 patients were confirmed to have cryptococcal infection [10]. In addition, detailed studies of cryptococcosis patients with other non-malignant hematological diseases, such as autoimmune hemolytic anemia (AIHA), immune thrombocytopenia (ITP), and Evans’ syndrome remain sparse [11]. Here, we retrospectively reviewed a series of cryptococcosis cases with hematological diseases admitted to our hospital from 2001 through 2014 to evaluate the possible risk factors, clinical characteristics, and prognosis of cryptococcosis in this population.

## Methods

### Patients and data resources

We retrospectively reviewed all proven and probable cases of cryptococcosis with hematological diseases admitted to Huashan Hospital, Fudan University (a tertiary health care center in Shanghai, China, with approximately 1200 hospital beds and 60,000 admissions per year) from January 2001 through December 2014. Patient demographics and other medical data were retrieved from the electronic medical record system in Huashan Hospital. Cases of cryptococcosis were identified using International Classification of Diseases (ICD) diagnostic codes. Individuals with one or more ICD-10 codes starting with ‘B45’ were classified as cryptococcosis cases. Medical records of these cases were carefully checked and patients were finally included in the cohort if a diagnosis of hematological diseases coexisted. None of the patients were HIV-infected. Clinical data of the enrolled patients was collected, including demographic information, underlying hematological diseases, other predisposing factors, clinical characteristics of cryptococcosis, and antifungal treatment.

### Definition of cryptococcosis

A proven diagnosis of cryptococcal meningitis was made if the patient met any of the following criteria: (1) positive culture of *Cryptococcus* from the cerebrospinal fluid (CSF), (2) positive CSF India ink smear, (3) positive cryptococcal capsular polysaccharide antigen in the CSF or (4) compatible histopathological findings, which comprise 5–10 μm encapsulated yeasts observed in the brain tissue. A proven diagnosis of pulmonary or sinus cryptococcosis was made through histopathology or tissue culture. A probable diagnosis of pulmonary or sinus cryptococcosis was made if all of the following conditions were met: (1) positive cryptococcal antigen titer in blood, (2) lesions of lung or sinus on computerized tomography (CT) scan, and (3) improvement of symptoms and radiology after antifungal treatment. Blood stream infection was confirmed if the blood culture of *Cryptococcus* was positive. Cryptococcal capsular polysaccharide antigen test of the CSF or serum was conducted using the latex-*Cryptococcus* antigen detection system (Immuno-Mycologics, Inc., USA). A result of at least 1:10 was considered positive [12, 13].

### Efficacy and safety assessment of antifungal therapy

Physicians chose antifungal therapy for the patients according to the Chinese guideline for cryptococcosis and available expert recommendations [14, 15]. Response to antifungal treatment in cryptococcosis patients was evaluated at the end of initial therapy on the basis of clinical, radiologic, and microbiologic data according to the consensus criteria issued by the European Organization for Research and Treatment of Cancer and Mycoses Study Group (EORTC/MSG) [16]. Efficacy of antifungal treatment was categorized as success (i.e., complete or partial response) or failure (i.e., stable response, disease progression, or death during the study period regardless of any cause). Patients with antifungal treatment less than 7 days were not included for efficacy evaluation. All-cause mortality at 2 weeks, 10 weeks and survival at 1-year follow-up were also analyzed. Adverse drug events (ADEs) of antifungal treatment were recorded. Relationship between ADEs and antifungal drugs was evaluated on the basis of the causality assessment system proposed by the World Health Organization Collaborating Centre for International Drug Monitoring, the Uppsala Monitoring Centre (WHO–UMC) [17].

### Statistical analysis

Summary statistics are expressed as mean ± standard deviations for continuous variables with normal distributions. Other data are expressed as median and range. Continuous variables within two groups were compared using the independent t-test for parametric data and the Mann-Whitney U test for non-parametric data. Categorical variables were expressed as proportions and compared using the Chi-square test or Fischer’s exact test, as appropriate. A *P*-value <0.05 was considered statistically significant. Statistical analysis was performed with the SPSS statistical package version 17.0 (SPSS, Inc., Chicago, USA).

## Results

### Demographic and clinical characteristics

**Fig. 1:**
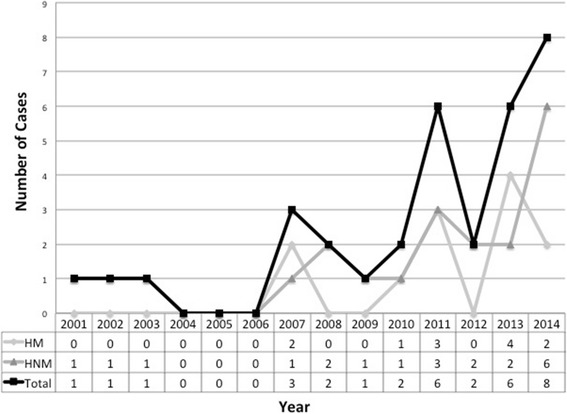
Distribution of cryptococcosis cases over 14 years. HM = hematological malignancies, HNM = hematological non-malignancies

During the study period, we identified 33 cryptococcosis patients with hematological diseases, among which 30 were proven and 3 were probable cases. Distribution of the 33 cases over 14 years is shown in Fig. 1. Demographic data and clinical characteristics of each patient were summarized in Table 1, and detailed information is listed in the Additional file 1. The median age of patients was 50 years (range, 16–79), and 42.4% were male. Twenty-one patients (63.6%) were diagnosed with non-malignant hematological diseases, including AIHA, Evans’ syndrome, and ITP. Twelve patients (36.4%) had confirmed hematological malignancies, including non-Hodgkin’s lymphoma (NHL), Waldenstrom’s macroglobulinemia (WM), acute lymphocytic leukemia (ALL), chronic lymphocytic leukemia (CLL), Hodgkin’s lymphoma (HL), multiple myeloma (MM), and myelodysplastic syndrome (MDS). The median time from the diagnosis of hematological diseases to the time of cryptococcosis was 73 weeks (ranging from 8 weeks to 15 years), except in 4 patients whose hematological diagnosis was confirmed after the onset of cryptococcosis. One patient was diagnosed with NHL 5 months after pulmonary cryptococcosis, one was diagnosed with WM 8 months after pulmonary cryptococcosis, another patient was diagnosed with AIHA 6 weeks after cryptococcal meningitis, and the last was diagnosed with NHL 3 months after cryptococcal meningitis diagnosis. Glucocorticoid usage was documented in 20 (20/21, 95.2%) of the non-malignancy patients. Usage of prednisone dosage higher than 0.3 mg/kg/d for more than 3 weeks was documented in 12 patients. A long-term use of low dose glucocorticoids was documented in 8 patients, and the duration of glucocorticoids use ranged from 2 months to more than 10 years. Eight among the 12 malignancy patients had undergone chemotherapy before infection. Prednisone was included in all chemotherapy regimens. Rituximab was used in 4 patients; among them one also received bevacizumab. Other predisposing factors for cryptococcosis included chronic kidney diseases in 2, splenectomy in 2, and rectal cancer in 1 patient.

**Table 1:** Summary of clinical characteristics of 33 cryptococcosis patients with hematological diseases

Variables	No. (%) of patients (*N* = 33)
Age, years	50 (range, 16–79)
Sex, female	19 (57.6)
Type of hematological diseases	
Hematological malignancies	12 (36.4)
NHL	4 (12.1)
WM	3 (9.1)
ALL	1 (3.0)
CLL	1 (3.0)
HL	1 (3.0)
MM	1 (3.0)
MDS	1 (3.0)
Non-malignant hematological disorders	21 (63.6)
AIHA	10 (30.3)
Evans’ syndrome	6 (18.2)
ITP	5 (15.1)
Other underlying conditions	
Chronic kidney diseases	2 (6.1)
Splenectomy	2 (6.1)
Rectal cancer	1 (3.0)
Chemotherapy	8 (24.2)
Glucocorticoids administration	28 (84.8)
Sites of cryptococcal infection^a^	
Brain	26 (78.8)
Lung	16 (48.5)
Blood	3 (9.1)
Sinus	2 (6.1)

*NHL* non-Hodgkin’s lymphoma; *WM* Waldenstrom’s macroglobulinemia; *ALL* acute lymphocytic leukemia; *CLL* chronic lymphocytic leukemia; *HL* Hodgkin’s lymphoma; *MM* multiple myeloma; *MDS* myelodysplastic syndrome; *AIHA* autoimmune hemolytic anemia; *Evans’* Evans’ syndrome; *ITP* immune thrombocytopenia

^a^Some of the patients had multi-site infection. Among 16 patients with lung infection, 9 had brain involvement as well. All patients with blood or sinus involvement had brain or lung infection at the same time

### Clinical manifestations

The sites of cryptococcal infections in patients with hematological diseases included brain in 26, lung in 16, blood in 3, and sinus in 2 patients. Some of the patients had multi-site infection. The main clinical manifestations among patients with central nervous system (CNS) infection were fever and headache (both were 24/26, 92.3%), as shown in Table 2. Sixteen of the febrile patients had temperatures higher than 39 °C, 5 patients were between 38.5-39 °C, and one patient had a maximum temperature of 38.2 °C. In addition, two patients were febrile but the temperature was not recorded. Vomiting (42.3%) was also commonly seen. Other symptoms included meningeal irritation, coma, epilepsy, hearing loss, blurred vision, and blindness. Sixteen patients had pulmonary cryptococcosis. Among them, 5 patients had cough, and 1 patient had shortness of breath. Respiratory symptoms were not observed in 11 other patients. Two patients with cryptococcal sinusitis presented with nasal congestion and pain.

**Table 2:** Clinical manifestations and laboratory findings of 33 patients

Variables	Patients with CNS involvement (*N* = 26)	Patients without CNS involvement (*N* = 7)
Manifestations, n (%)		
Fever	24 (92.3)	1 (14.3)
Headache	24 (92.3)	0
Vomiting	11 (42.3)	0
Meningeal irritation signs	8 (30.8)	0
Coma	7 (26.9)	0
Epilepsy	7 (26.9)	0
Hearing loss	3 (11.5)	0
Blurred vision	2 (7.7)	0
Cough	2 (7.7)	3 (42.9)
Shortness of breath	0	1 (14.3)
Asymptomatic	0	4 (57.1)
Other symptoms^a^	3 (11.5)	0
Baseline CSF parameters		
Opening Pressure, mmH2O^b^	>300 (120, >300)	113 (75, 260)
WBC, cells/mm^3c^	28 (1, 533)	2 (1, 7)
Protein, g/L^d^	0.77 (0.18, 2.6)	0.28 (0.16, 0.73)
Glucose, mmol/L^e^	1.6 (0.14, 5.32)	3.2 (2.8, 3.9)
CrAg titer^f^	1:1280 (1:640, >1:1280)	All negative
Indian ink smear, +/−^g^	23/2	0/7
Culture for *Cryptococcus*, +/−^h^	17/3	0/7
Baseline serum CrAg titer^i^	1:1280 (1:640, 1:5120)	1:10 (Negative, 1:640)

*CNS* central nervous system; *CSF* cerebrospinal fluid; *WBC* white blood cell count; *CrAg* cryptococcal antigen

^a^Other symptoms included blindness in 1, loss of recent memory in 1 and symptoms of brain stem herniation in 1

^b^CSF opening pressure was not documented in 5 CNS infected patients and 2 CNS uninfected patients

^c^CSF WBC was not documented in 6 CNS infected patients

^d^CSF protein was not documented in 4 CNS infected patients

^e^CSF glucose was not documented in 4 CNS infected patients

^f^CSF CrAg was not documented in 5 CNS infected patients. In 1 patient, CSF CrAg was positive, but titer was not recorded

^g^CSF Indian ink smear was not documented in 1 CNS infected patient

^h^CSF culture for *Cryptococcus* was not documented in 6 CNS infected patients

^i^Serum CrAg titer was not documented in 11 CNS infected patients

### Laboratory findings

Lumbar punctures at baseline were performed in all 33 patients. Most of the CNS infected patients had elevated opening pressure, CSF cell count and protein level, as well as decreased CSF glucose concentration. CSF culture was positive in 17 (85%) out of 20 patients, while 23 out of 25 (92%) had positive India ink smears for *Cryptococcus*. CSF cryptococcal antigen test results were documented in 20 patients, with all being positive and ranging from 1:640 to over 1:1280. Moreover, CSF parameters were normal in patients without CNS infection as summarized in Table 2. Among the 33 patients included, serum cryptococcal antigen titer was documented in 22 cases. Nineteen of them were positive, ranging from 1:10 to over 1:1280. Three patients, whose antigen titers in blood were negative, had a confirmed diagnosis of pulmonary cryptococcosis through histopathology. Serum titers equal or greater than 1:640 were observed more frequently in CNS infected patients than those without CNS infection (15/15 vs. 1/7, χ2 = 13.866, *P* < 0.001). Blood culture analyzed in 12 patients for *Cryptococcus* showed 3 to be positive (25%).

### Image findings

Abnormal cranial findings were presented in 18 out of 25 (72%) meningitis patients either by CT scan or magnetic resonance imaging (MRI). Common sites of lesions were frontal lobe in 7, parietal lobe in 5, basal ganglion in 6, periventricular region in 6, temporal lobe in 2, occipital lobe in 2, and cerebellum in 1 patient. Some patients had multi-site involvement. Lesions were characterized by decreased density in the CT scan. MRI usually revealed low signal in T1 and high signal in T2, with or without enhancement. Abnormalities of chest CT were found in all 16 patients with pulmonary infections. The most common findings were single or multi nodules or masses (14/16). Patchy airspace consolidation and cavities were described in 7 and 2 patients respectively. Pleural effusion was only documented in one patient. Some patients had more than two types of lesions on the chest CT scan.

### Treatment and outcomes

All patients received antifungal treatment. Among the 26 cryptococcal meningitis patients, 18 were initially treated with amphotericin B (AmB) based therapy. Amphotericin B deoxycholate (d-AmB) daily dosage ranged from 20 to 30 mg (0.35–0.59 mg/kg/d, median 0.43 mg/kg/d). Median of accumulated d-AmB dosage was 1425 mg and ranged from 106 to 7400 mg. Note that liposomal AmB (L-AmB) was used in 2 patients with a daily dosage of 50 mg, and total dose of 850 mg and 1425 mg, respectively. As shown in Table 3, at the end of initial therapy 10 out of 18 (55.6%) patients achieved clinical success (all were partial response), 4 (22.2%) patients had stable response, one patient had progression of disease and 2 patients died. Fluconazole-based initial therapy was administered to 5 CNS-infected patients. One patient died on the first day of antifungal treatment and was not included in the efficacy assessment. None of the remaining 4 patients achieved clinical success (all showed stable response). Three more patients received initial therapy with voriconazole or itraconazole, but only one patient showed a partial response. Three patients were given an additional d-AmB intrathecal injection. Surgical intervention was performed in 2 patients, one with external ventricular drainage and another with ventriculoperitoneal shunt. The median duration of total antifungal therapy among these 26 patients was 18 weeks, ranging from one day to 160 weeks. Three patients died within the first 2 weeks of antifungal therapy (3/26, 11.6%) and five patients died within 10 weeks (5/25, 20.0%). Survival rate at 1-year follow-up was 72.0% (18/25). One patient each were lost in the 10-week and 1-year follow-up. These data are summarized in Table 3.

**Table 3:** Initial antifungal therapy and outcomes of 26 patients with CNS involvement

Variables	No. (%) of patients (*N* = 26)
Initial antifungal therapy	
AmB based	18 (69.2)
Fluconazole based	5 (19.2)
Other^a^	3 (11.6)
Efficacy of initial antifungal therapy	
Success	11 (42.3)
Complete response	0
Partial response	11 (42.3)
Failure	15 (57.7)
Stable	10 (38.5)
Progression	1 (3.8)
Death	4 (15.4)
Outcome^b^	
2-week mortality rate	3/26 (11.6%)
10-week mortality rate	5/25 (20.0%)
1-year survival rate	18/25 (72.0%)

*CNS* central nervous system; *AmB* amphotericin B

^a^Other initial antifungal therapies included voriconazole in 2, itraconazole in 1 patient

^b^One patient lost for 10-week and 1-year follow-up

For patients without CNS involvement, 5 patients were treated with fluconazole, while the other two were treated with d-AmB as THE initial therapy (total dosage of 500 mg). The median time of antifungal therapy duration was also 18 weeks and ranged from 3 to 27 weeks. Clinical response for pulmonary cryptococcosis was 85.7% (6/7). Pulmonary lobectomy was performed in one patient. Only one patient died of hepatic failure (drug-unrelated) after 4 weeks of fluconazole-based therapy. In pulmonary cryptococcosis patients, 2-week and 10-week mortality was 0% and 14.3%, respectively. One-year survival rate was 85.7%.

Twelve out of 18 CNS-infected patients who received AmB had drug-related ADEs, including hypokalemia (4 certain cases), thrombocytopenia (4 cases with 2 certain, 1 probable and 1 possible), leukopenia (1 certain case), renal function abnormalities (1 certain case, 1 possible case), and liver function abnormalities (1 possible case). Three patients were switched from AmB-based therapy to other therapies; two of them due to thrombocytopenia, and the reason for the third patient was not documented. Flucytosine was added in 18 patients as part of the initial therapy. Two of them withdrew flucytosine due to allergy (possible) and leukopenia (possible), respectively. No ADEs were reported during fluconazole treatment.

### Comparison between patients with hematological malignancies and non-malignancies

In total, there were 12 patients with hematological malignancies and 21 with non-malignancies (Table 4). The patients in these two groups showed no significant differences in age or gender. Chemotherapy was more frequently administered to patients with hematological malignancies (8/12 vs. 0/21, *P* < 0.001), while more patients with non-malignancies received glucocorticoids (20/21 vs. 8/12, *P* = 0.047). Most non-malignant hematological patients (20/21, 95.2%) had CNS infection. In contrast, only six (6/12, 50.0%) patients with hematological malignancies had CNS involvement (χ2 = 9.351, *P* = 0.001).

**Table 4:** Comparison of clinical characteristics between patients with hematological malignancies and non-malignancies (*N* = 33)

Variables	Patients with hematological malignancies (%) (*N* = 12)	Patients with hematological non-malignancies (%) (*N* = 21)	*P* value
Age, years	52.5 (16–79)	50 (20–77)	0.586
Sex, female	3 (25.0)	16 (76.2)	0.126
Non-hematological underlying conditions			
Chronic kidney diseases	0 (0)	2 (9.5)	0.523
Splenectomy	0 (0)	2 (9.5)	0.523
Rectal cancer	0 (0)	1 (4.8)	1.000
Chemotherapy	8 (66.7%)	0 (0)	<0.0001
Glucocorticoids administration	8 (66.7%)	20 (95.2%)	0.047
Sites of cryptococcal infection			
Brain	6 (50.0)	20 (95.2)	0.001
Lung	8 (66.7)	8 (38.1)	0.157
Blood	1 (8.3)	2 (9.5)	1.000
Sinus	1 (8.3)	1 (4.8)	1.000

In cryptococcal meningitis patients, clinical characteristics such as manifestation, laboratory and radiology findings, treatment and outcomes were also compared between cases with hematological malignancies and non-malignancies (Table 5). Vomiting was more common in the hematological non-malignancies group (11/20 vs. 0/6, *P* = 0.024). However, no significant differences were found in other items.

**Table 5:** Comparison of manifestations, laboratory finding, clinical response of antifungal treatment, outcomes between cryptococcal meningitis patients with hematological malignancies and non-malignancies

Variables	CM Patients with hematological malignancies (%) (*N* = 6)	CM Patients with hematological non-malignancies (%) (*N* = 20)	*P* value
Symptoms			
Fever	5 (83.3)	19 (95.0)	0.415
Headache	6 (100.0)	18 (90.0)	1.000
Vomitting	0 (0.0)	11 (55.0)	0.024
Radiology of brain^a^			
Multisite lesions, Yes/No	5/1	10/8	0.351
Parenchyma damage, Yes/No	5/1	13/5	1.000
Baseline CSF parameters			
Opening Pressure > 250mmH_2_O, Yes/No^b^	3/2	10/6	1.000
WBC, cells/mm^3c^	31.5 (20, 40)	34.5 (1, 533)	0.802
Protein, g/L^d^	0.74 (0.184, 1.015)	0.74 (0.277, 2.601)	0.773
Glucose <1.1 mmol/L, Yes/No^e^	4/1	13/4	1.000
Indian ink smear, +/−^f^	6/0	17/2	1.000
Culture for *Cryptococcus*, +/−^g^	3/1	14/2	0.509
CrAg titer >1:1280, Yes/No^h^	1/5	5/9	0.613
Baseline serum CrAg titer >1:1280, Yes/No^i^	4/2	5/8	0.350
Treatment			
AmB-based therapy	4 (66.7)	14 (70.0)	1.000
Success	3 (50.0)	8 (40.0)	1.000
Outcome			
2-week mortality	0 (0.0)	3 (15.0)	1.000
10-week mortality^j^	1 (16.7)	4 (21.1)	1.000

^a^Image findings of the brain was not documented in 2 patients with hematological non-malignancies

^b^CSF opening pressure was not documented in 1 patient with hematological malignancies and 4 with non-malignancies

^c^CSF WBC was not documented in 2 patient with hematological malignancies and 4 with non-malignancies

^d^CSF protein was not documented in 1 patient with hematological malignancies and 3 with non-malignancies

^e^CSF glucose was not documented in 1 patient with hematological malignancies and 3 with non-malignancies

^f^CSF Indian ink smear was not documented in 1 patient with hematological non-malignancies

^g^CSF culture for *Cryptococcus* was not documented in 2 patient with hematological malignancies and 4 with non-malignancies

^h^CSF CrAg was not documented in 6 patients with hematological non-malignancies

^i^Serum CrAg titer was not documented in 7 patients with hematological non-malignancies

^j^One patient with hematological non-malignancies was lost for 10-week follow-up

## Discussion

In previous studies, hematological neoplasis was the most common concomitant conditions in patients with hematological diseases, among which lymphoma was most frequently reported [18–21]. Only one study showed that leukemia was the most common underlying hematological malignancy in cryptococcosis patients [22]. Our study reviewed 33 cryptococcosis cases predisposed to hematological diseases in a 14-year span and showed that lymphoma (41.7%) was the most frequently encountered malignancy. Notably, only a few cases of cryptococcosis have been reported in patients with non-malignant hematological conditions. A recently published literature review summarized the clinical features of cryptococcal meningitis in 5 patients with AIHA and 8 previously reported other cases [11]. Additionally, in Pappas’s large study on HIV-negative cryptococcosis, 29 out of 306 (9%) subjects had concomitant hematological malignancies, while no cases with hematological non-malignant conditions were reported [23]. In Lee’s 18-year study on cryptococcal meningitis, 21.6% had predisposing hematological and oncological conditions; however, a detailed breakdown was not given [24]. In our study, majority (21/33) of the patients had hematological non-malignant conditions. AIHA was the most common one, accounting for 47.6% of non-malignancies and followed by ITP and Evans’ syndrome. Considering these data, it is likely that cryptococcosis may be underestimated among this subpopulation.

Brain, lung and blood were undoubtedly found to be the most common infection sites. The majority of cryptococcosis cases in our series had CNS involvement, which is much higher than the description by Tseng in non-HIV patients (53.7%) and similar to that in HIV conditions (81.5%) [25]. Clinical manifestations of patients were unspecific [26, 27]. The most common symptoms of CNS infection in our study were fever, headache, and vomiting. In addition, many of these patients had temperature over 38.5 °C, which is consistent with a previous report that immunocompromised patients were more likely to have high fever [28]. Identification of the infecting pathogen was performed through CSF culture or India ink smear, which are traditional methods for the diagnosis of cryptococcal meningitis. Almost all of the CNS-infected patients (25/26) were confirmed by either positive culture or smear. In addition to these methods, cryptococcal antigen test is an important addition to the diagnostics and is widely used [29]. In cryptococcal meningitis patients, the positivity of CSF cryptococcal antigen test could be up to 100%, indicating that this test should be conducted in suspected CNS-involved patients as well as the CSF culture and smear. Patients with only lung involvement were either asymptomatic, or only had mild cough. Abnormal lesions in the lung can be the only clue for cryptococcal infection. This would make early diagnosis of these cases more difficult, and perhaps may increase the possibility of CNS dissemination. Thus, cryptococcal antigen test of serum are needed for differential diagnosis. In this study, we observed that the serum cryptococcal antigen titer in CNS-infected patients was relatively higher (equal or higher than 1:640). This is consistent with that in HIV-infected conditions more than that in HIV-uninfected ones [25]. In patients with only pulmonary involvement, 4 were positive for cryptococcal antigen in blood and 3 were negative. The proportion with higher titers in blood was lower in these patients than that in cryptococcal meningitis. Similar findings were reported in a previous study, which showed that infection in the lung resulted in lower cryptococcal antigen and a higher incidence of negative serum antigen tests [26]. As a result, lung biopsy should be conducted when pulmonary cryptococcosis is suspected with a negative antigen test; indeed, we confirmed the pulmonary cryptococcosis diagnosis in 3 of our patients using this method.

Treatment of cryptococcal meningitis in the setting of hematological diseases is difficult, due to the impaired immunity, frequent ADEs, and the lack of supporting evidence for this small population. The Infectious Diseases Society of America (IDSA) guideline provides a general recommendation of d-AmB with or without flucytosine therapy for non-HIV patients however, the regimen for patients with hematological disorders was not specified [30]. Based on recently published guideline for CNS infections in patients with hematological disorders (including hematological malignancies and allogeneic stem-cell transplantation), L-AmB or AmB lipid complex (ABLC) should be the first choice for cryptococcal meningitis as drug related bone marrow suppression and hemolysis are of great concern [31]. In our case series, most patients were treated with d-AmB-based initial therapy according to the Chinese guideline for cryptococcosis at a dosage of 0.35–0.59 mg/kg/d (median 0.43 mg/kg/d), and the majority of them also received flucytosine (100 mg/kg/d). Clinical responses were achieved in most of them, consistent with our previous study in HIV-uninfected patients with cryptococcal meningitis [28]. Both d-AmB and flucytosine were well tolerated in our series, suggesting that d-AmB with or without flucytosine could be a safe and effective choice for treatment of cryptococcal meningitis in hematological patients. For patients without CNS infection, fluconazole with or without flucytosine was used. In this group, most patients tolerated fluconazole well and had a favorable response.

Previous studies on cryptococcal infection in hematological settings in general have emphasized the malignant conditions. However, we observed that non-malignant hematological patients seemed more likely to develop CNS infection than malignancy patients. The high proportion of long-term corticosteroids administration in non-malignant patients, which could lead to infection dissemination, may in part, be responsible for this difference. Furthermore, in the fatal cryptococcal meningitis, the two groups of patients did not differ significantly in other baseline characteristics such as symptoms, laboratory findings, treatment response, and outcomes. Therefore, cryptococcal infection, especially CNS involvement should not be neglected in hematological non-malignant patients.

To our knowledge, this is the largest case series of cryptococcosis patients with hematological diseases to date. However, our study has several limitations. It is a single center-based study in a tertiary care hospital, so that the number of cases we enrolled was small. All cases in our cohort were HIV-uninfected; thus, our experience may not be representative of those with HIV-infection. Meanwhile, due to the retrospective nature of our study design, some of the clinical data are incomplete, which decreased the power of statistical analysis. Larger, multicenter studies are needed to fully identify the characteristics of this population. Moreover, randomized controlled trials should be conducted to compare the different antifungal regimens to provide specific evidence for clinical practice in the future.

## Conclusions

Cryptococcal infection has been known to be uncommon in patients with hematological diseases and thus can be easily misdiagnosed. In addition to commonly identified hematological malignant conditions, non-malignant conditions are also risk factors for cryptococcal infection and have a tendency for CNS involvement. Symptoms of cryptococcal infection in hematological patients were similar with other patient groups and were unspecific, suggesting the importance of cryptococcal antigen test, and CSF culture or smear. D-AmB based initial treatment could be a choice for cryptococcal meningitis patients with hematological diseases, while most patients with only lung involvement could be treated with fluconazole.

## Additional file

Additional file 1: Table S1. Detailed clinical characteristics of 33 cryptococcosis patients with hematological diseases. (DOC 86 kb)

## Abbreviations

ABLC: amphotericin B lipid complex
ADEs: adverse drug events
AIHA: autoimmune hemolytic anemia
ALL: acute lymphocytic leukemia
AmB: amphotericin B
CLL: chronic lymphocytic leukemia
CNS: central nervous system
CSF: cerebrospinal fluid
CT: computerized tomography
d-AmB: amphotericin B deoxycholate
EORTC/MSG: Mycoses Study Group and European Organization for Research and Treatment of Cancer
HIV: human immunodeficiency virus
HL: Hodgkin’s lymphoma
HSCT: hematopoietic stem cell transplantation
ICD: International Classification of Diseases
IDSA: Infectious Diseases Society of America
ITP: immune thrombocytopenia
L-AmB: liposomal amphotericin B
MDS: myelodysplastic syndrome
MM: multiple myeloma
MRI: magnetic resonance imaging
NHL: non-Hodgkin’s lymphoma
WBC: white blood cell
WHO–UMC: World Health Organization Collaborating Centre for International Drug Monitoring, the Uppsala Monitoring Centre
WM: Waldenstrom’s macroglobulinemia
