# Supplementary material for: Cryptococcosis in patients with hematological diseases: a 14-year retrospective clinical analysis in a Chinese tertiary hospital
Source: BMC Infect Dis. 2017 Jul 3;17:463. doi: 10.1186/s12879-017-2561-z (PMC5496217; doi:10.1186/s12879-017-2561-z)
Supplement: Additional file 1: Table S1. — Detailed clinical characteristics of 33 cryptococcosis patients with hematological diseases. (DOC 86 kb) [file 12879_2017_2561_MOESM1_ESM.doc]

| **Table S1. Detailed clinical characteristics of 33 cryptococcosis patients with hematological diseases** | | | | | | | | | | |  |
| --- | --- | --- | --- | --- | --- | --- | --- | --- | --- | --- | --- |
| **Patient NO.** | **Hematological disease** | **Sites of infection** | **Cerebrospinal fluid** | | | | | **Blood** | | |  |
|  |  |  | **WBC (/mm^3^)** | **Protein**  **(g/L)** | **Glucose**  **(mmol/L)** | **Antigen titer** | **Culture/Smear** | | **Antigen titer** | **Culture** | |
| P1 | AIHA | B | 52 | 0.95 | 2.61 | 1:1280 | +/+ | | 1:1280 | NA | |
| P2 | AIHA | B | NA | >0.93 | <1.11 | NA | +/- | | NA | NA | |
| P3 | AIHA | B | NA | 0.8 | 1.52 | >1:1280 | +/- | | >1:1280 | NA | |
| P4 | AIHA | B | 3 | 0.35 | 4.7 | 1:640 | -/+ | | 1:1280 | NA | |
| P5 | AIHA | B | NA | NA | NA | + | NA/+ | | NA | NA | |
| P6 | AIHA | B | 8 | 1.13 | 0.14 | 1:1280 | NA/+ | | NA | NA | |
| P7 | AIHA | B, L | 177 | 0.66 | 2.31 | 1:1280 | +/+ | | NA | NA | |
| P8 | AIHA | B | 60 | NA | 1 | >1:1280 | +/+ | | >1:1280 | - | |
| P9 | AIHA | B, Bl | 1 | 0.47 | 1.4 | 1:640 | +/+ | | >1:1280 | + | |
| P10 | AIHA | L | 7 | 0.728 | 3.2 | - | -/- | | 1:320 | - | |
| P11 | Evans’ | B | 10 | 0.71 | NA | 1:1280 | +/- | | 1:640 | NA | |
| P12 | Evans’ | B | 110 | 0.874 | 2 | >1:1280 | +/+ | | 1:640 | - | |
| P13 | Evans’ | B | 115 | 0.74 | 2 | 1:1280 | +/- | | 1:1280 | - | |
| P14 | Evans’ | B, L | NA | NA | NA | NA | +/+ | | 1:1280 | NA | |
| P15 | Evans’ | B, L | 41 | 0.44 | 0.97 | NA | +/+ | | >1:1280 | - | |
| P16 | Evans’ | B, L, | 21 | 0.28 | 5.32 | NA | +/+ | | NA | - | |
| P17 | ITP | B | 20 | 0.47 | 1.9 | >1:1280 | NA/+ | | NA | NA | |
| P18 | ITP | B, L | 533 | 1.97 | 4.06 | NA | +/+ | | NA | NA | |
| P19 | ITP | B, L, Bl | 7 | 2.6 | 1.1 | 1:1280 | NA/NA | | 1:1280 | + | |
| P20 | ITP | B, L | 56 | 0.85 | 0.32 | 1:5120 | -/+ | | 1:2560 | NA | |
| P21 | ITP | B, S | 28 | 0.48 | 1.57 | 1:1280 | +/+ | | 1:1280 | NA | |
| P22 | NHL | B | 40 | 0.184 | 0.75 | 1:1280 | +/+ | | 1:640 | NA | |
| P23 | NHL | B, S | NA | NA | NA | 1:1280 | N/+ | | >1:1280 | - | |
| P24 | NHL | L | 7 | 0.25 | 2.8 | - | -/- | | - | NA | |
| P25 | NHL | L | 1 | 0.16 | 2.8 | - | -/- | | 1:640 | NA | |
| P26 | WM | B | 28 | 0.74 | 1.1 | 1:1280 | -/+ | | >1:1280 | NA | |
| P27 | WM | B | 20 | 0.4 | 1.6 | >1:1280 | +/+ | | >1:1280 | - | |
| P28 | WM | L | 4 | 0.25 | 3.2 | - | -/- | | - | NA | |
| P29 | ALL | L, Bl | 2 | 0.49 | 3.9 | - | -/- | | >1:1280 | + | |
| P30 | CLL | B, L | 35 | 1.02 | <0.56 | 1:1280 | +/+ | | >1:1280 | - | |
| P31 | HL | L | 2 | 0.33 | 2.8 | - | -/- | | 1:10 | - | |
| P32 | MM | L | 2 | 0.275 | 3.4 | - | -/- | | - | - | |
| P33 | MDS | B, L | NA | >1 | <2.1 | 1:1280 | NA/+ | | 1:1280 | NA | |

P = patient, F = female, M = male, B = brain, L = lung, S = sinus, Bl = blood, NA = not available

AIHA = autoimmune hemolytic anemia, Evans’ = Evans’ syndrome, ITP = immune thrombocytopenia, NHL = non-Hodgkin’s lymphoma,

WM = Waldenstrom's macroglobulinemia, ALL = acute lymphocytic leukemia, CLL = chronic lymphocytic leukemia, HL = Hodgkin’s lymphoma,

MM = multiple myeloma, MDS = myelodysplastic syndrome
